# Role of miR-142-3p in the Post-Transcriptional Regulation of the Clock Gene *Bmal1* in the Mouse SCN

**DOI:** 10.1371/journal.pone.0065300

**Published:** 2013-06-05

**Authors:** Vikram R. Shende, Nichole Neuendorff, David J. Earnest

**Affiliations:** 1 Department of Biology, Texas A&M University, College Station, Texas, United States of America; 2 Center for Biological Clocks Research, Texas A&M University, College Station, Texas, United States of America; 3 Department of Neuroscience and Experimental Therapeutics, Texas A&M Health Science Center, College of Medicine, Bryan, Texas, United States of America; Florida State University, United States of America

## Abstract

MicroRNAs (miRNAs) are small non-coding RNAs that function as post-transcriptional modulators by regulating stability or translation of target mRNAs. Recent studies have implicated miRNAs in the regulation of mammalian circadian rhythms. To explore the role of miRNAs in the post-transcriptional modulation of core clock genes in the master circadian pacemaker, we examined miR-142-3p for evidence of circadian expression in the suprachiasmatic nuclei (SCN), regulation of its putative clock gene target *Bmal1* via specific binding sites in the 3′ UTR and overexpression-induced changes in the circadian rhythm of BMAL1 protein levels in SCN cells. In mice exposed to constant darkness (DD), miR-142-3p levels in the SCN were characterized by circadian rhythmicity with peak expression during early subjective day at CT 3. Mutagenesis studies indicate that two independent miRNA recognition elements located at nucleotides 1–7 and 335–357 contribute equally to miR-142-3p-induced repression of luciferase-reported *Bmal1* 3′ UTR activity. Importantly, overexpression of miR-142-3p in immortalized SCN cells abolished circadian variation in endogenous BMAL1 protein levels *in vitro*. Collectively, our results suggest that miR-142-3p may play a role in the post-transcriptional modulation of *Bmal1* and its oscillatory regulation in molecular feedback loops mediating SCN circadian function.

## Introduction

In mammals, the suprachiasmatic nuclei (SCN) of the anterior hypothalamus function as the master pacemaker mediating the generation and light-dark entrainment of circadian rhythms [1], [2]. In addition to coordinating circadian rhythmicity in other brain regions and peripheral tissues, the SCN is characterized by ensemble and cell-autonomous circadian oscillations in many of its cellular and molecular processes independent of external input [3]–[7]. These endogenous oscillations are especially prevalent in the expression of genes comprising the molecular clockworks and are thus vital to the circadian oscillator and pacemaking functions of the SCN. The circadian clock mechanism common to both SCN and peripheral cells consists of feedback interactions between brain, muscle ARNT-like protein 1 (*Bmal1*), circadian locomotor output cycles kaput (*Clock*), as well as the period (*Per1* and *Per2*) and cryptochrome (*Cry1* and *Cry2*) genes in which the transcription of these core molecular components is rhythmically regulated by their protein products with exception of *Clock* [8]–[13].

While post-translational processes including phosphorylation, ubiquitination, sumoylation, and acetylation have garnered the most attention for their role in the regulation of circadian feedback loops [14], [15], recent studies have revealed that post-transcriptional mechanisms are also involved in the modification of clock proteins and oscillatory behavior of core molecular components [16], [17]. In this regard, emerging evidence suggests that microRNAs (miRNAs) may contribute to the post-transcriptional modulation of circadian clock function. Mature miRNAs are small non-coding RNAs, usually 19–25 nucleotides in length, and their interactions with miRNA-recognition elements (MRE’s) in the 3′ untranslated regions (UTR) of target genes induce mRNA de-stabilization and/or translational repression [18]–[21]. The potential role of miRNAs in SCN clock control of circadian rhythms was first observed in experiments indicating that miR-219 and miR-132 expression in the SCN oscillates in a circadian manner and antagonism of these miRNAs respectively alters the circadian period and light-induced phase shifts of the activity rhythm in mice [22]. It is noteworthy that miRNAs have also been implicated in the modulation of peripheral circadian clocks as antisense inhibition of miR-122 has been shown to induce post-transcriptional perturbations in the circadian expression of many genes involved in hepatic lipid and cholesterol metabolism [23].

Despite the increasing focus on miRNA function in the regulation of mammalian circadian rhythms, limited information is available on whether specific miRNAs target key components of the molecular clockworks and contribute to the oscillatory regulation of clock gene transcripts. In *Drosophila*, *bantam* encodes a miRNA that has been shown to regulate the translation of *clock* via interactions with multiple target sites within the 3′ UTR of this gene [24]. In mammals, the miR-192/194 cluster has been identified as a potent repressor of the 3′ UTRs of all *Per* genes [25]. Because miR-142-3p is distinguished by robust modulation of *Bmal1* 3′ UTR activity in mammalian peripheral oscillators [26] and by the presence of a canonical, CACGTG E-box element in its promoter region that may provide for clock control of miR-142 transcription, the present study focused on the role of this miRNA in the post-transcriptional regulation of *Bmal1* in the master circadian clock within the SCN. The objectives of our experimental analysis were to determine whether: **1**) miR-142-3p is rhythmically expressed in the SCN *in vivo* and in an immortalized SCN cell line; **2**) the repression of *Bmal1* 3′ UTR activity in response to miR-142-3p overexpression is abated by mutagenesis of specific miRNA binding sites; and **3**) miR-142-3p overexpression affects the endogenous BMAL1 protein rhythm in SCN cells *in vitro*.

## Materials and Methods

### Experiment 1: Temporal Profiling of miR-142-3p Expression in SCN cells *in vivo* and *in vitro*

#### Animals and SCN tissue collection

Experimental subjects were 40 male C57BL/6J mice at 6–8 weeks of age (JAX Mice & Services, Bar Harbor, ME). Animals were maintained in the vivarium at Texas A&M University System Health Science Center under a standard 12h light: 12 h dark cycle (LD 12∶12; lights-on at 0600 h). Animals were housed 4–5 per cage with *ad libitum* access to food and water, and periodic animal care was performed at random times. All procedures used in this study were approved by the University Laboratory Animal Care Committee at Texas A&M University.

To determine whether miR-142-3p expression fluctuates rhythmically in the SCN *in vivo*, mice were maintained in LD 12∶12 for 3 weeks prior to experimental analysis and then exposed to constant darkness (DD) beginning at lights-off in the LD 12∶12 cycle (1800 h). Beginning 15 hours later (0900 h or circadian time [CT] 3), animals were sacrificed at 4 h intervals (*n* = 5) for 24 h by decapitation using an infrared viewer (FJW Optical Systems, Palatine, IL). SCN tissue was immediately dissected as described previously [27], [28]. All tissue samples were frozen in liquid nitrogen and stored at −80°C until further processing.

For *in vitro* analysis, immortalized SCN cell lines generated from *mPer2^Luc^* knockin and from mice with targeted disruption of *Per1* and *Per2* (*Per1^ldc/^Per2^ldc^*) were used to profile the temporal pattern of miR-142-3p expression. These cell lines were maintained and propagated as described previously [29]. Briefly, cells were grown on laminin-coated 60 mm dishes (Corning, Inc.) in Minimal Essential Medium (MEM; Invitrogen, Grand Island, NY) containing 10% Fetal Bovine Serum (FBS), 3000 µg/ml glucose and 292 µg/ml L-glutamine. Fresh medium was applied every 48 h and cultures were split 1∶3 to 1∶5 every 3–4 days. Prior to experimentation, cells were expanded onto laminin-coated 6-well plates (BD Biosciences, San Jose, CA). Approximately 24 h after plating, the medium was changed so as to reduce the FBS concentration to 5% and on the following day cells were rinsed with calcium-magnesium free (CMF) phosphate-buffered saline and then cultured in serum-free Neurobasal medium containing B27 supplement (1X, Invitrogen). Cultures (n = 5) were harvested at 4 h intervals for 36 h by trypsinization and cell pellets were flash frozen in liquid nitrogen. All samples were stored at −80°C until subsequent analysis of miRNA or mRNA content.

#### RNA extraction and Real-time PCR

Total cellular RNA was later extracted from individual mouse SCN tissue samples and cultures of *mPer2^Luc^* and *Per1^ldc^*/*Per2^ldc^* SCN cells using miRNeasy kit (Qiagen, Inc., Valencia, CA) according to the manufacturer’s protocols. Total RNA was estimated using a Nanodrop ND2000 (Thermo Scientific, Rockford, IL). Quantitative real-time PCR analysis for miR-142-3p was conducted using Taqman microRNA assays (Applied Biosystems) as described previously [26]. Briefly, miR-142-3p from individual samples was reverse transcribed using Taqman MicroRNA Reverse Transcription Kit and the cDNA equivalent of 1.5 ng of total RNA was PCR amplified in an ABI PRISM 7500 Fast sequence detection system using the following standard conditions: **1**) heating at 95°C for 10 min, and **2**) amplification over 40 cycles at 95°C for 15 sec and 60°C for 1 min. As an endogenous control for differences in sample RNA content and reverse-transcription efficiencies, U6 snRNA was also amplified from the same samples using identical parameters. Using the comparative C_T_ method described in the ABI Prism 7700 Sequence Detection System User Bulletin #2 (PE-ABI), the relative abundance of miR-142-3p was calculated by normalization first to corresponding U6 snRNA levels in each sample and then to a calibrator consisting of pooled cDNA from multiple samples over the entire time series.

Relative quantification of *Bmal1* mRNA abundance in all samples was performed using SYBR-Green real-time PCR technology (ABI) as described previously [30], [31]. To generate single-strand cDNAs, total RNA (1 µg) from individual samples was reverse transcribed using random hexamers and Superscript III reverse transcriptase Kit (Invitrogen). Real-time PCR analysis was performed on duplicate aliquots using the cDNA equivalent of 1 ng of total RNA for each sample. The PCR cycling conditions were: **1**) serial heating at 50°C for 2 min and 95°C for 10 min, **2**) amplification over 40 cycles at 95°C for 15 sec and 60°C for 1 min, and **3**) dissociation at 95°C for 15 sec, 60°C for 1 min, 95°C for 15 sec and 60°C for 15 sec. To control for differences in sample RNA content, cyclophilin A (*Ppia*) was amplified with the cDNA equivalent of 1 ng total RNA from the same samples. Consistent with our previous studies using this gene in a similar manner [31], [32], *Ppia* showed no sign of circadian or ultradian variation (data not shown). The comparative C_T_ method was similarly applied to determine the relative expression of *Bmal1* mRNA.

The following primers were used for real-time PCR analysis:

*Bmal1* forward: 5′- CCAAGAAAGTATGGACACAGACAAA-3′;

*Bmal1* reverse: 5′- GCATTCTTGATCCTTCCTTGGT-3′;

*Ppia* forward: 5′- TGTGCCAGGGTGGTGACTT-3′;

*Ppia* reverse: 5′- TCAAATTTCTCTCCGTAGATGGACTT-3′.

### Experiment 2: Mutagenic Analysis of Putative Binding Sites Mediating miR-142-3p-induced Repression of *Bmal1* 3′ UTR Activity

#### Bmal1 3′ UTR luciferase reporter constructs

miTarget™ miRNA Target Sequence 3′ UTR Expression Clone containing *Bmal1* 3′ UTR sequence (Accession: NM_007489.3) inserted in the pEZX-MT01 vector was purchased from GeneCopoeia, Inc (Rockville, MD). The plasmid was propagated using methods established in our previous study [26]. Deletions in predicted miR-142-3p binding sites on the *Bmal1* 3′ UTR were generated using QuikChange II XL Site-Directed Mutagenesis Kit (Stratagene, La Jolla, CA) according to the manufacturer’s protocols. Briefly, the full-length *Bmal1* 3′ UTR was PCR mutagenized using specific primers so as to delete nucleotides 1–7 complementary to miR-142-3p seed region. After *Dnp1-*mediated degradation of parental plasmid DNA, the mutagenized plasmid was transformed into XL10-Gold Ultracompetent cells (Stratagene) and transformants were selected on kanamycin-containing (final conc.  = 50 µg/ml) imMedia agar plates (Invitrogen). A single colony was isolated and propagated in imMedia Kan^+^ liquid medium (final conc.  = 50 µg/ml). The plasmid was extracted using HiSpeed Plasmid Midi Kit (Qiagen, Inc.) and then sequenced to verify the targeted deletion (*Bmal1 c.1_7del)*. Identical methods were also used to delete nucleotides 335–357 corresponding to a second predicted miR-142-3p binding site on the *Bmal1* 3′ UTR complementary to the seed region along with additional nucleotides that may function as a 3′ supplementary or compensatory element and aid in miRNA biological activity [33]–[35]. The resulting plasmid (*Bmal1 c.335_357del*) was then subjected to a second round of mutagenesis to provide for additional deletion of nucleotides 1–7. The plasmid with targeted deletions of both miR-142-3p target sites on the *Bmal1* 3′ UTR (*Bmal1 c.1_7del*+*c.335_357del*) was propagated and sequenced to verify these deletions as described above. The miRNA 3′ UTR target control vector (Genecopoeia; CmiT000001-MT01), consisting of the pEZX-MT01 vector backbone without any 3′ UTR sequence, was used to determine the specificity of miRNA interactions with the full-length and mutagenized *Bmal1* vectors. miR-142-3p-mediated regulation of *Bmal1* 3′ UTR was analyzed in human embryonic kidney (HEK293) cells using established methods [26]. Briefly, cells were seeded onto 24-well plates (Corning, Inc., Tewksbury MA) and 24 h later were co-transfected with pEZX-MR04 miR-142 expression vector (miExpress Precursor miRNA expression clone; Genecopoeia, Inc., MmiR3437-MR04) and with either the target control, full-length *Bmal1* 3′ UTR (WT), *Bmal1 c.1_7del, Bmal1 c.335_357del* or *Bmal1 c.1_7del*+*c.335_357del* miRNA 3′ UTR target clones. As an additional control for specificity of the deletion, cells were also co-transfected with miR-494 expression vector and either the target control, *Bmal1* or *Bmal1 c.1_7del+c.335_357del* miRNA 3′ UTR target clones. Following transfection for 5 hours, cells were rinsed and growth medium was replaced. Forty-eight hours later, cell lysates of HEK293 cultures from all treatment groups (n = 4) were prepared using Passive Lysis Buffer (Promega Corp., Madison, WI). Firefly and *Renilla* luciferase activity from individual samples was then analyzed using the dual-luciferase reporter assay system (Promega Corp.). Luminescence was measured on Synergy2 microplate luminometer (BioTek, Winooski, VT) and reported as the ratios of firefly luciferase signal normalized to *Renilla* luciferase activity in the same sample.

### Experiment 3: Effects of miR-142-3p Overexpression on the Circadian Regulation of *BMAL1* Protein Levels in SCN cells *in vitro*

The effects of miR-142-3p overexpression on endogenous levels of mBMAL1 protein were examined in cultures of *mPer2^Luc^* SCN cells that were derived from a single passage. *mPer2^Luc^* SCN cells were expanded on laminin-coated 60-mm dishes (Corning, Inc.) maintained at 37°C and 5% CO2 in MEM (Invitrogen, Inc.) supplemented with 3000 µg/ml D-glucose, 292 µg/ml L-glutamine and 10% Fetal Bovine Serum (Hyclone). Prior to experimentation, cells were seeded onto laminin-coated 6-well plates and 24 h later, cultures were transfected with pEZX-MR04 miRNA expression vector for miR-142 (final conc.  = 4.0 µg/well) using Lipofectamine 2000 (Invitrogen) according to the manufacturer’s guidelines. Control cultures were similarly transfected with a pEZX-MR04 vector encoding a scrambled, non-targeting control sequence. Based on preliminary observations derived from fluorescence microscopy examining the expression of the eGFP reporter encoded in both pEZX-MR04 vectors (miR-142 expression and scrambled, non-targeting control), the transfection efficiency in *mPer2^Luc^* SCN cells ranged from 40–50%. Following transfection for 5 h, cells were rinsed and cultured in growth medium supplemented with 5% FBS. On the following day, cells were rinsed with CMF buffer and then maintained in serum-free Neurobasal medium containing B27 supplement (1X, Invitrogen). Beginning 12 h later, control- and miR-142-3p-transfected cultures (n = 5) were harvested at 4 h intervals for 24 h by trypsinization and cell pellets were flash frozen in liquid nitrogen. All samples were stored at −80°C until subsequent processing. Cell pellets were sonicated in mammalian protein extraction reagent (MPER; Pierce Biotechnology, Inc., Rockford, IL) supplemented with protease inhibitor cocktail (PMSF), and protein content in cell homogenate samples was measured using the bicinchoninic acid method (BCA Protein Assay Kit; Thermo Scientific Pierce).

#### Western blotting

BMAL1 protein levels in SCN cell lysates were assessed by Western blot analysis using the XCell SureLock™ Mini-cell and Novex Western Transfer Apparatus (Invitrogen). Samples were loaded at ∼20 µg protein per lane onto 10% Tris-Glycine gels. Following separation at 125 V for 2 h, proteins were transferred onto 0.45 µm nitrocellulose membranes (Invitrogen) and blocked at room temperature for 1 hour with 5% skimmed milk in Tris-buffered saline (TBS) supplemented with 0.1% Tween-20 (TBS-T). With interceding rinses in TBS-T, membranes were probed overnight at 4°C with rabbit anti-BMAL1 (1∶250; Abcam, Cambridge, MA) or a mouse monoclonal antibody against β-actin (1∶1000; Sigma-Aldrich Corp., St. Louis, MO) followed by a 1-hour incubation with horse-radish peroxidase (HRP)-conjugated donkey anti-rabbit IgG (1∶1000) or goat anti-mouse IgG (1∶2000; Jackson ImmunoResearch Laboratories, Inc., West Grove, PA). Immunoreactive signal for BMAL1 was generated using enhanced chemiluminescence (ECL) reagent (Thermo Scientific Pierce) and luminescence for size-appropriate bands was detected using a FluorChem Gel imaging system (Alpha Innotech Corp., San Leandro, CA). To control for differences in protein content between samples, signal intensity measurements for BMAL1 were normalized to the values for β-actin in each sample. Using NIH ImageJ software, densitometric analyses for immunoreactive bands were performed on data derived from five biological replicates and two technical replicates.

#### Statistical analyses

Circadian fluctuations in miR-142-3p and *Bmal1* mRNA expression were identified by cosine curve-fitting analysis using GraphPad Prism software. With α = 0.05, waveform fittings with p-values ≤0.01 were considered statistically significant and therefore characterized by circadian variation. Paired comparisons between peak and trough values were analyzed *post hoc* for statistical differences using the Student Newman-Keuls sequential range test. The α-value was set at 0.05 for these *post hoc* analyses.

Independent *t*-tests were performed on normalized luciferase bioluminescence data to determine the significance of miRNA-mediated repression of luciferase-reported *Bmal1* 3′ UTR activity. The α-value was set at 0.05 for all independent t-tests. Temporal patterns of BMAL1 protein expression were first analyzed by one-way analysis of variance (ANOVA). Paired comparisons between peak values and those observed during the preceding or succeeding minimum were analyzed *post hoc* for statistical differences using the Student Newman-Keuls sequential range test. The α-value was set at 0.05 for these *post hoc* analyses.

## Results

### Experiment 1: Temporal Profiling of miR-142-3p Expression in SCN cells *in vivo* and *in vitro*

Prior to determining whether miR-142-3p expression in SCN cells fluctuates on a circadian basis, we analyzed the promoter region of this miRNA for evidence of E-box or CRE-box elements which are known to mediate clock- and light-controlled transcription, respectively. The presence of a canonical, CACGTG E-box element was identified ∼1.5 Kb upstream of the miR-142 locus and analysis of syntenic regions indicated that this regulatory element is highly conserved among mammalian lineages.

**Figure 1:**
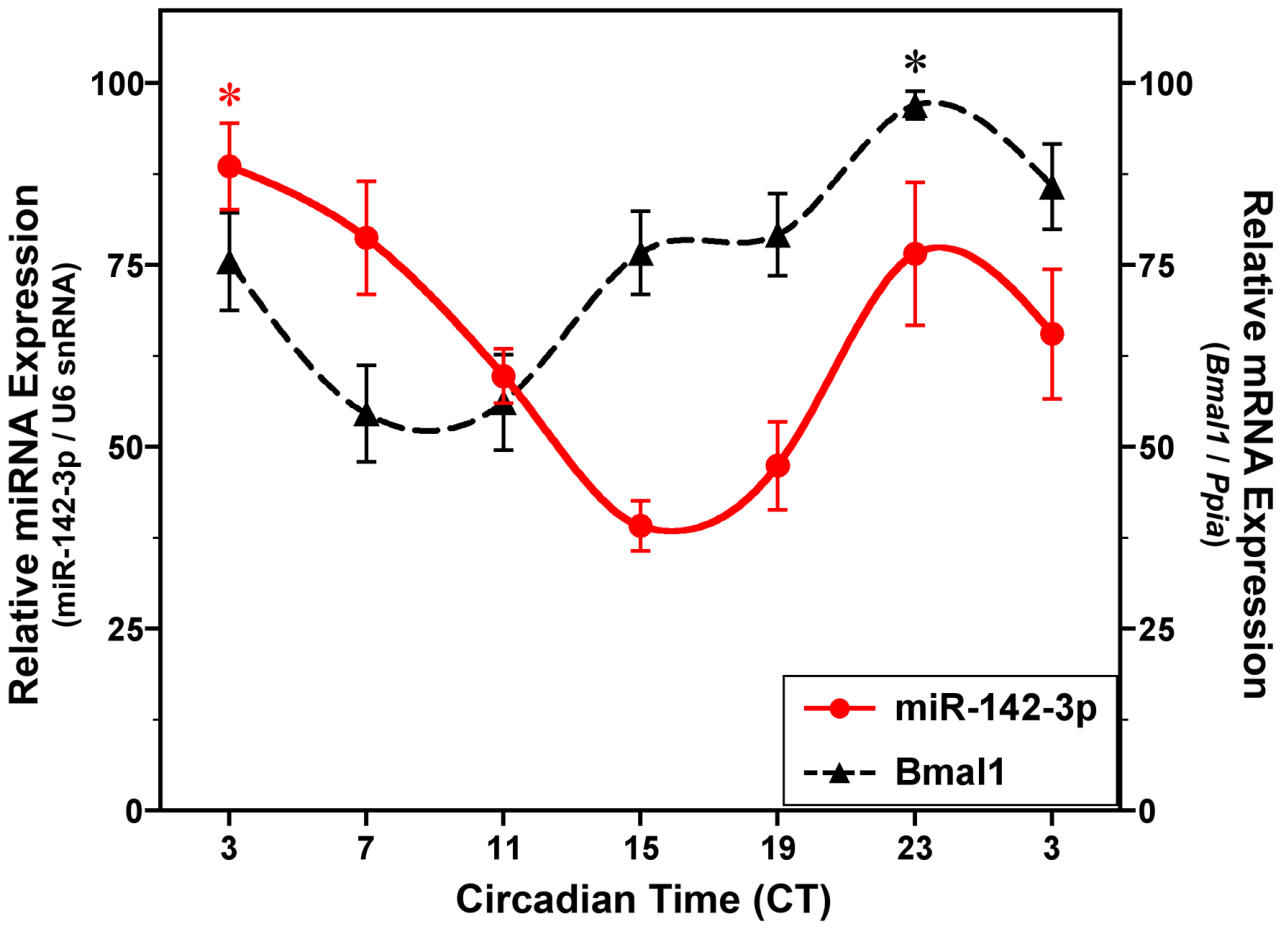
Temporal patterns of miR-142-3p and *Bmal1* expression in the mouse SCN. Symbols denote real-time PCR determinations (mean ± SEM) of miR-142-3p (red) and *Bmal1* mRNA (black) levels at 4-hour intervals in the SCN (n = 4-5) of mice exposed to constant darkness (DD). The plotted values correspond to the ratios of miR-142-3p signal normalized to U6 snRNA levels and of *Bmal1*/*Ppia* mRNA signal in which the maximal value for each gene was set at 100%. Asterisks indicate time points during which peak levels were significantly greater (*p*<0.05) than those observed during preceding or succeeding minima.

Because identification of an E-box element in the promoter region of miR-142 suggests that its transcription may be clock-controlled, we next examined the temporal profile of miR-142-3p expression in the SCN *in vivo*. During exposure to DD, the relative abundance of miR-142-3p (normalized to U6 snRNA) in the mouse SCN was marked by circadian variation (*p*<0.01) with peak expression occurring early in subjective day and low levels throughout the early and middle portions of the subjective night (Fig. 1). The rhythmic peak in SCN expression of miR-142-3p at CT 3 was significantly (*p*<0.05) and about 2-fold greater than the succeeding minima observed during the early subjective night at CT 15. Parallel analysis of *Bmal1* expression in the same samples revealed that SCN content of this clock gene mRNA was characterized by a circadian profile (*p*<0.01) similar to that reported previously [36], [37]. The peak in SCN expression of *Bmal1* mRNA during the late subjective night at CT 23 was significantly greater (*p*<0.01) than that observed during the preceding minima. The phase relationship between circadian oscillations in *Bmal1* mRNA and miR-142-3p expression in the mouse SCN is noteworthy; the rising phase and peak of the *Bmal1* rhythm occurred in advance of peak miR-142-3p expression in the SCN, raising the possibility that *Bmal1* may play a role in the activation of miR-142-3p transcription.

**Figure 2:**
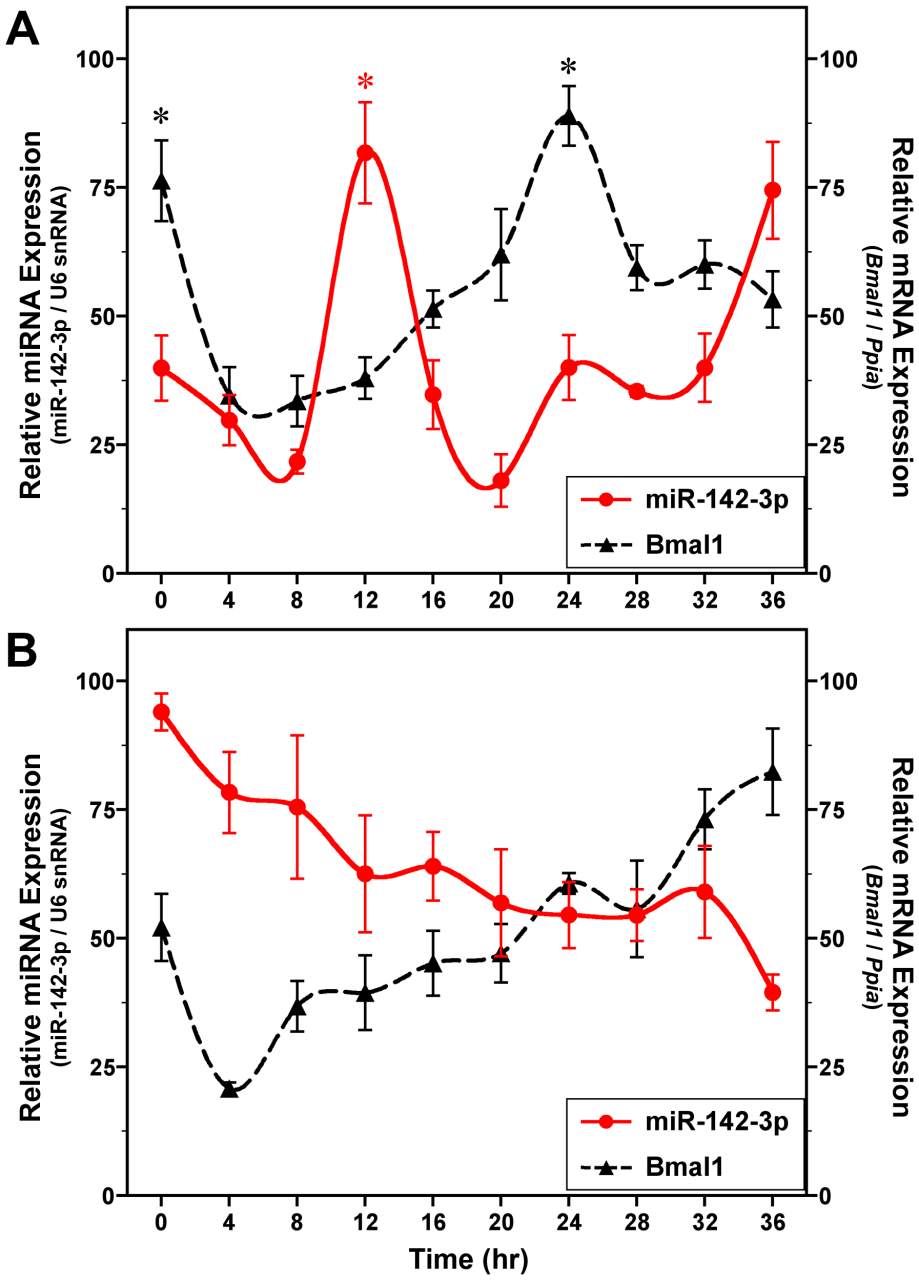
Temporal patterns of miR-142-3p and *Bmal1* expression in immortalized SCN cells *in vitro*. Symbols denote real-time PCR determinations (mean ± SEM) of miR-142-3p (red) and *Bmal1* mRNA (black) levels at 4-hour intervals in cultures (n = 4–5) of *mPer2^Luc^* SCN cells (**A**) and immortalized SCN cells (*Per1^ldc^/Per2^ldc^*) with targeted disruption of *Per1* and *Per2* (**B**). The plotted values correspond to the ratios of miR-142-3p signal normalized to U6 snRNA levels and of *Bmal1*/*Ppia* mRNA signal in which the maximal value for each gene was set at 100%. Asterisks indicate time points during which peak levels were significantly greater (*p*<0.05) than those observed during preceding or succeeding minima.

Studies using immortalized *mPer2^Luc^* SCN cells were subsequently conducted to determine whether levels of mature miR-142-3p are also rhythmically regulated *in vitro* because this cell line has been shown to retain the endogenous oscillatory properties of the SCN *in vivo* (ensemble rhythms of Farnell et al. [29]). Similar to the temporal profile observed in the mouse SCN, miR-142-3p expression (normalized to U6 snRNA) oscillated with a circadian periodicity (*p*<0.01) in cultures of *mPer2^Luc^* SCN cells (Fig. 2A). The amplitude of this circadian rhythm in miR-142-3p levels was robust, with 3- to 4-fold differences between peak and trough values. The rhythmic peak of miR-142-3p expression in *mPer2^Luc^* SCN cells at 12 h was significantly greater (*p*<0.01) than the minima observed at 20 h. Circadian variation in *Bmal1* expression was also observed in the same *mPer2^Luc^* SCN cultures (*p*<0.01), with circadian peaks in *Bmal1* mRNA levels at 0 h and 24 h that were significantly greater (*p*<0.01) than the nadir observed at 8 h. The *Bmal1* and miR-142-3p rhythms in *mPer2^Luc^* SCN cells were marked by overt phase differences such that the oscillation in *Bmal1* mRNA levels was antiphasic to the circadian profile in miR-142-3p expression (Fig. 2A). This phase relationship between *Bmal1* mRNA and miR-142-3p rhythms in SCN cells *in vitro* differed from that observed in the SCN *in vivo*. Although the basis for the phase differences between *in vivo* and *in vitro* SCN rhythms is unknown, an important consideration is that analysis of the mouse SCN was conducted following stable entrainment to a strong synchronizing signal (i.e., LD cycle) whereas experiments with *mPer2^Luc^* SCN cultures were performed in the absence of advance exposure to a defined phase-resetting stimulus (e.g., serum shock).

To verify that the observed miR-142-3p rhythm in SCN cells is under control of the circadian clock, the temporal profile of miR-142-3p expression was analyzed in a SCN cell line derived from mice with targeted disruption of *Per1* and *Per2* genes (*Per1^ldc^*/*Per2^ldc^*). These mutant mice have been shown to exhibit an arrhythmic behavioral phenotype [38] and our previous findings indicate that SCN-specific circadian pacemaking function is similarly abolished in immortalized *Per1^ldc^*/*Per2^ldc^* SCN cells [29]. *Bmal1* mRNA levels in *Per1^ldc^*/*Per2^ldc^* SCN cultures fluctuated over time but this variation was not characterized by circadian or other rhythmic harmonics. MiR-142-3p expression in the same cultures remained largely at constant levels and showed no significant evidence of circadian rhythmicity (*p*>0.05) (Fig. 2B), indicating that the miR-142-3p oscillation observed in immortalized SCN cells *in vitro* is indeed clock-controlled.

### Experiment 2: Mutagenic Analysis of Putative Binding Sites Mediating miR-142-3p-induced Repression of *Bmal1* 3′ UTR Activity

**Figure 3:**
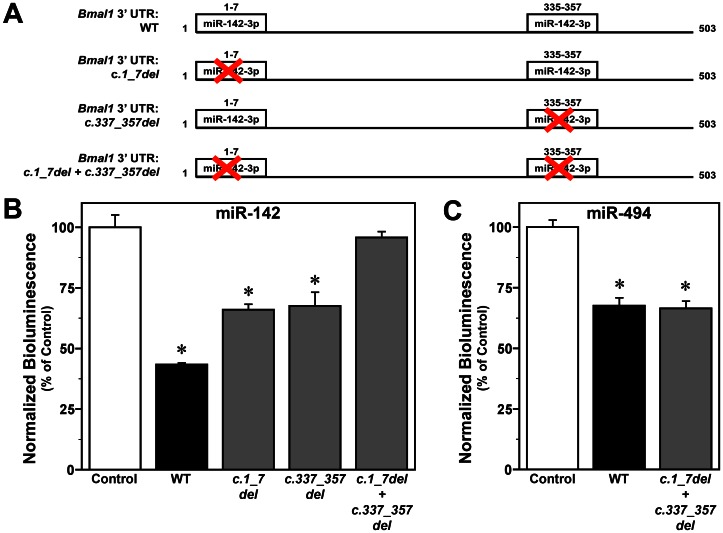
Effects of binding site mutagenesis on miR-142-3p-induced repression of *Bmal1* 3′ UTR activity. (A) Diagrammatic representation of WT and mutagenized *Bmal1* 3′ UTR constructs with independent site-directed deletions of predicted miR-142-3p binding sites. (B) Normalized bioluminescence from HEK293 cells co-transfected with pEZX-MR04 miR-142 expression vector and with either the target control, full-length *Bmal1* (WT), *Bmal1 c.1_7del, Bmal1 c.335_357del* or *Bmal1 c.1_7del*+*c.335_357del* miRNA 3′ UTR target clones. (C) Normalized bioluminescence from HEK293 cells co-transfected with pEZX-MR04 miR-494 expression vector with either the target control, full-length *Bmal1* (WT), *Bmal1 c.1_7del, Bmal1 c.335_357del* or *Bmal1 c.1_7del*+*c.335_357del* miRNA 3′ UTR target clones. Bars represent mean (±SEM) determinations of luciferase bioluminescence for each treatment group (n = 4). The plotted values correspond to the ratios of firefly luciferase signal normalized to *Renilla* luciferase activity in the same sample and are represented as a percentage of the average signal for control vector transfectants. Asterisks denote comparisons in which normalized bioluminescence for a given treatment group was significantly reduced (*p*<0.05) relative to that observed in control vector transfectants.

Our previous findings indicate that miR-142-3p represses *Bmal1* 3′ UTR activity [26]. Based on target prediction algorithms, this miR-142-3p-mediated repression is predicted to occur via two consensus binding sites on the *Bmal1* 3′ UTR. To evaluate the specificity and relative contribution of these predicted miR-142-3p binding sites, we analyzed luciferase-reported *Bmal1* 3′ UTR activity in HEK293 cells co-transfected with the pEZX-MR04 miR-142 expression vector and constructs for either full-length *Bmal1* 3′ UTR (WT) or mutants with independent site-directed deletions of nucleotides 1–7 (*c.1_7del)* or nucleotides 335–357 which includes the second seed region and putative 3′ supplementary or compensatory elements (*c.335_357del*) or combined deletions of both loci (*c.1_7del*+*c.335_357del*) (Fig. 3A). Consistent with our previous results [26], miR-142-3p overexpression produced significant decreases (*p*<0.01) of ∼60% in luciferase-reported bioluminescence in WT *Bmal1* 3′ UTR-expressing HEK293 cells relative to that found in cells transfected with the control vector (Fig. 3B). In HEK293 cells co-transfected with either the *c.1_7del* or *c.335_357del Bmal1* 3′ UTR, overexpression of miR-142-3p similarly induced a significant (*p*<0.05) reduction in luciferase-mediated bioluminescence relative to control transfections but the amplitude of this effect was diminished (∼30%) in comparison to that observed for the full-length *Bmal1* 3′ UTR. Combined deletion of both 3′ UTR target sites (*c.1_7del*+*c.335_357del*) completely abolished repression of the *Bmal1* 3′ UTR in response to miR-142-3p-overexpression as no significant differences in bioluminescence (*p = 0.33*) were observed in cells transfected with control vector or the *c.1_7del*+*c.335_357del* construct (Fig. 3B). Collectively, these findings indicate that both of the putative miR-142-3p binding sites contribute equally to the repression of the *Bmal1* 3′ UTR.

In addition to miR-142-3p, miR-494 has also been shown to repress *Bmal1* 3′ UTR activity [26], potentially by interacting with nucleotides 473-495. Consequently, we examined the effects of miR-494 on full-length and mutant (*c.1_7del*+*c.335_357del*) *Bmal1* 3′ UTR activity to verify the specificity of the miR-142-3p binding sites at nucleotides 1–7 and 335–357. In HEK293 cells co-transfected with the *c.1_7del*+*c.335_357del* 3′ UTR construct, overexpression of miR-494 induced a significant reduction (*p*<0.01) of ∼35% in luciferase-reported bioluminescence relative to that found in cells transfected with the control vector (Fig. 3C). Importantly, this miR-494-mediated repression of the *Bmal1* 3′ UTR with combined deletion of the putative miR-142-3p binding sites was similar to that observed for the full-length 3′ UTR construct, suggesting that both mutagenized loci are specific targets for the action of miR-142-3p.

### Experiment 3: Effects of miR-142-3p Overexpression on the Circadian Regulation of *BMAL1* Protein Levels in SCN cells *in vitro*

**Figure 4:**
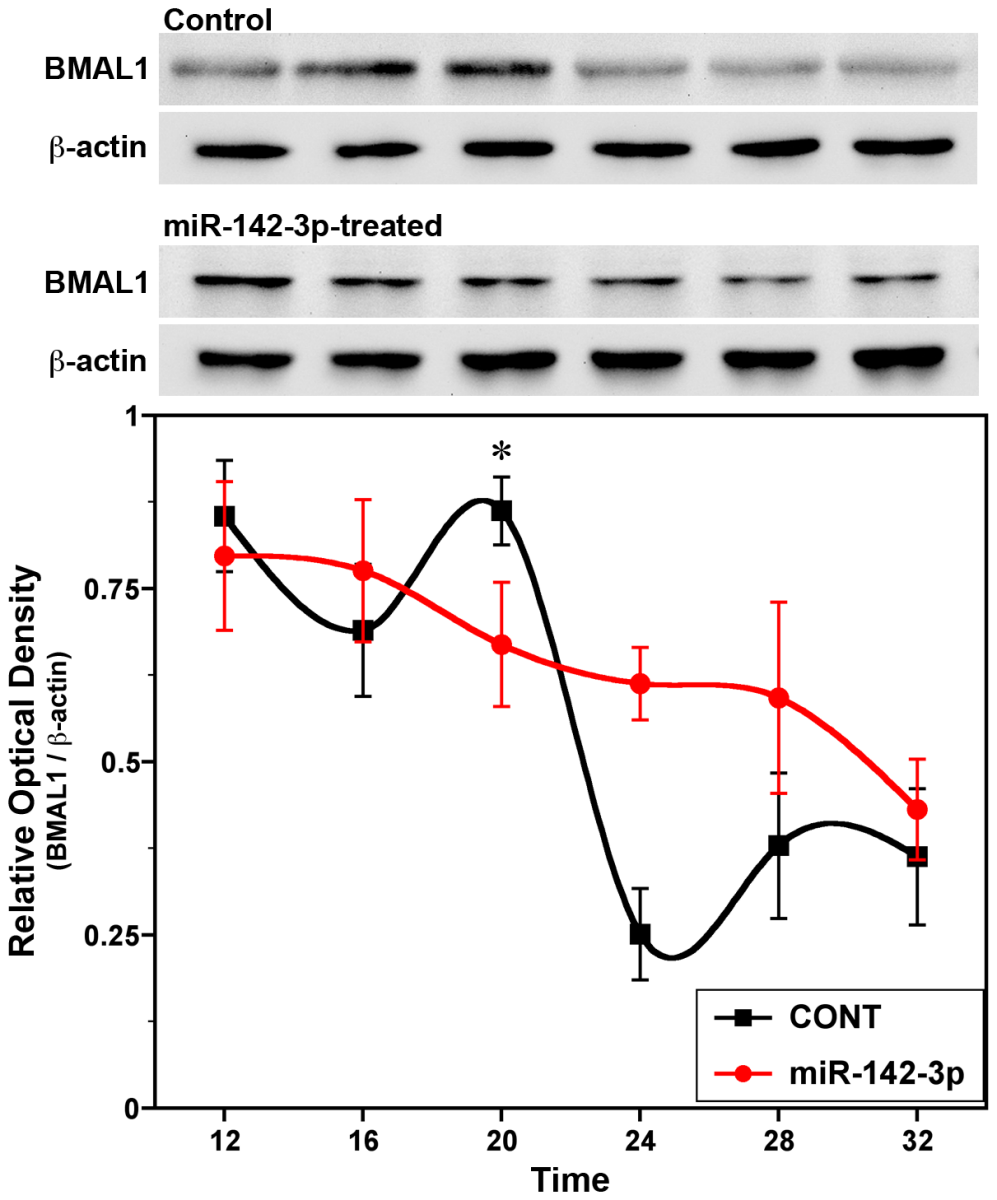
Effects of miR-142-3p overexpression on the circadian rhythm of BMAL1 protein content in immortalized SCN cells *in vitro*. Representative Western blot results and densitometric determinations of BMAL1 protein levels at 4-hour intervals in cultures (n = 4–5) of *mPer2^Luc^* SCN cells transfected with pEZX-MR04 control miRNA expression vector (**CONT**) or pEZX-MR04 miR-142 expression vector (**miR-142**). The plotted values represent the relative optical density (mean ± SEM) and correspond to the ratios of BMAL1/β-actin immunoreactive signal in each sample. The asterisk indicates that the peak in BMAL1 protein levels at 20 hr was significantly greater (*p*<0.05) than that observed during succeeding minima.

To investigate functional implications of miR-142-3p in the regulation of SCN clock gene oscillations, we next determined whether constitutive overexpression of miR-142-3p in immortalized *mPer2^Luc^* SCN cells affects the circadian rhythm in endogenous BMAL1 protein levels. In cultured *mPer2^Luc^* SCN cells transfected with the scrambled control vector, BMAL1 content varied significantly (*p*<0.001) over time (Fig. 4). The rhythmic peak in BMAL1 protein levels at 20 h was significantly (p<0.01) and about 3-fold higher than the succeeding trough observed 8 h later. In comparison with control cultures, the BMAL1 rhythm was abolished in miR-142-3p-transfected *mPer2^Luc^* SCN cultures as content of this clock gene protein remained at intermediate levels with no significant variation (p>0.05). The disruption of rhythmicity induced by miR-142-3p overexpression was associated with a significant decrease (p<0.05) in BMAL1 protein levels at 20 h relative to peak values observed at this timepoint in control cultures.

## Discussion

In the SCN, circadian oscillations in many of the core clock genes as well as clock-controlled genes are essential to the autonomous oscillator properties of individual cells and their ensemble function as the master circadian pacemaker in mammals. Recent evidence indicates that miRNAs play a role in the post-transcriptional processes that mediate the oscillatory properties and timekeeping function of core clock genes. In NIH/3T3 fibroblasts, overexpression of the miR-192/194 cluster represses the 3′ UTRs of *Per1*, *Per2* and *Per3* and shortens the circadian period of the *Bmal1* mRNA rhythm [25]. The current study provides the first evidence for the role of miRNAs in the regulation of specific clock genes and their cyclical modulation in the master pacemaker of mammalian circadian rhythms. Similar to many of its endogenous biological processes, SCN expression of miR-142-3p fluctuates rhythmically and circadian regulation of this miRNA is dependent on the integrity of the molecular clockworks. In addition, miR-142-3p modulates *Bmal1* expression in the mouse SCN and plays a role in the circadian control of this clock gene as over-expression abolishes the rhythm in BMAL1 protein accumulation. Because *Bmal1* is widely expressed and rhythmically regulated in most cells and tissues throughout the body [39], miR-142-3p may play a similar modulatory role in the post-transcriptional regulation of core molecular components in peripheral clocks.

The phase relationship between miR-142-3p and *Bmal1* rhythms in the SCN is compatible with our evidence for the function of this miRNA as a post-transcriptional repressor of *Bmal1*. In the SCN, miR-142-3p levels reached peak values during the early subjective day when *Bmal1* expression was low. In conjunction with evidence that miR-142-3p is a bona-fide clock-controlled gene, the localization of a conserved, canonical E-box (CANNTG) element ∼1.5 kb upstream of the miR-142 locus suggests that its clock gene target may feed back and positively regulate the transcription of this miRNA through the formation of CLOCK-BMAL1 heterodimer complexes. Based on the observation that CLOCK-BMAL1 abundance fluctuates in the mouse SCN with peak levels occurring at CT 0 [37], it appears that the putative timing of these positive transcriptional regulatory complexes is appropriately phased in advance of the zenith in SCN miR-142-3p expression at CT 3. Relative to other miRNA-target relationships, miR-142-3p and *Bmal1* are thus unique because the miRNA represses its target gene but the target also drives expression of the miRNA.

In mammals, the activity of miRNAs as post-transcriptional repressors is primarily dependent on conserved complementarity between 3′ UTR elements of the target mRNA and 7-8mer sites in the seed region comprising nucleotides 2–8 of the miRNA [33], [40], [41]. In the *Bmal1* 3′ UTR, nucleotides 1–7 are complementary to seed region of miR-142-3p. Consistent with the predicted significance of seed region interactions in functional mRNA–miRNA pairing, deletion of the first seven nucleotides in the *Bmal1* 3′ UTR abated miR-142-3p-mediated repression by ∼50%. In addition to this portion of the 3′ UTR, deletion of a highly conserved, canonical miRNA recognition element (MRE) at nucleotides 335–357 encompassing an octamer complementary to the seed region of miR-142-3p also yielded a comparable reduction (∼50%) in the repressive effect of this miRNA on *Bmal1* 3′ UTR activity. Site-directed deletion of both putative MREs at nucleotides 1–7 and 335–357 completely abolished miR-142-3p-induced, but not miR-494-mediated, inhibition of *Bmal1* 3′ UTR activity. Thus, these observations provide primary evidence indicating that two independent MREs on the *Bmal1* 3′ UTR are responsible for binding miR-142-3p and both contribute equally to its modulatory effects on 3′ UTR activity. The high degree of conservation for miR-142-3p and the two MREs on the *Bmal1* 3′ UTR suggest that miR-142-3p may contribute to the feedback modulation of *Bmal1* and perhaps other core elements of the molecular clockworks in the SCN across different mammalian species.

Because miRNAs function as molecular switches controlling expression of hundreds of genes [42], [43], the impact of miR-142-3p and its circadian modulation may extend beyond the regulation of *Bmal1*. In this regard, miR-142-3p has also been shown to modulate cAMP levels by targeting adenylate cyclase 9 in T-cells [43], [44]. In the context of the present study, the possible modulation of this second messenger by miR-142-3p is intriguing because cAMP levels in the rat SCN fluctuate on a circadian basis with bimodal peaks during the late subjective day and late subjective night [45] and cAMP pathways are involved in regulating the SCN circadian rhythms [45], [46]. Hence, miR-142-3p may play a role in the circadian physiology of the SCN by regulating not only the core clock gene, *Bmal1*, but also clock-controlled outputs like cAMP so as to provide feedback capable of resetting and/or fine-tuning the clock mechanism.
